# Effect of germination environment on the biochemical compounds and anti-inflammatory properties of soybean cultivars

**DOI:** 10.1371/journal.pone.0232159

**Published:** 2020-04-27

**Authors:** Hyang Lan Eum, Yeri Park, Tae Gyu Yi, Jae Wook Lee, Keon-Soo Ha, Ik-Young Choi, Nam Il Park

**Affiliations:** 1 Department of Plant Science, Gangneung-Wonju National University, Gangneung, Republic of Korea; 2 Natural Product Research Center, Korea Institute of Science and Technology, Gangneung, Republic of Korea; 3 Gangwondo Agricultural Research and Extension Services, Chuncheon, Republic of Korea; 4 Department of Agriculture and Life Industry, Kangwon National University, Chuncheon, Republic of Korea; Institute for Biological Research, SERBIA

## Abstract

In this study, we investigated changes in the isoflavone content, total phenolic content (TPC), total flavonoid content (TFC), antioxidant activities (DPPH, ABTS), and anti-inflammatory activities of small-seeded and large-seeded soybean cultivars during germination (light/dark conditions). Total isoflavone content was higher at the seed stage in large-seeded soybeans, while it increased after 7 days of germination in small-seeded soybeans, particularly in response to light conditions, under which they had high TPC, TFC, and antioxidant activities. In large-seeded soybeans, the germination environment did not significantly affect TFC or DPPH inhibition, whereas TPC and ABTS inhibition were high under dark germination conditions. Extracts of sprouts exhibited superior anti-inflammatory activities. Nitric oxide production was slightly lower in small-seeded and large-seeded soybeans germinated under light and dark conditions, respectively. Our findings indicate that germinated soybeans improved nutritionally, and that enhancement of bioactivity under different germination environments could contribute to the selection of appropriate soybean cultivars.

## Introduction

**Fig 1:**
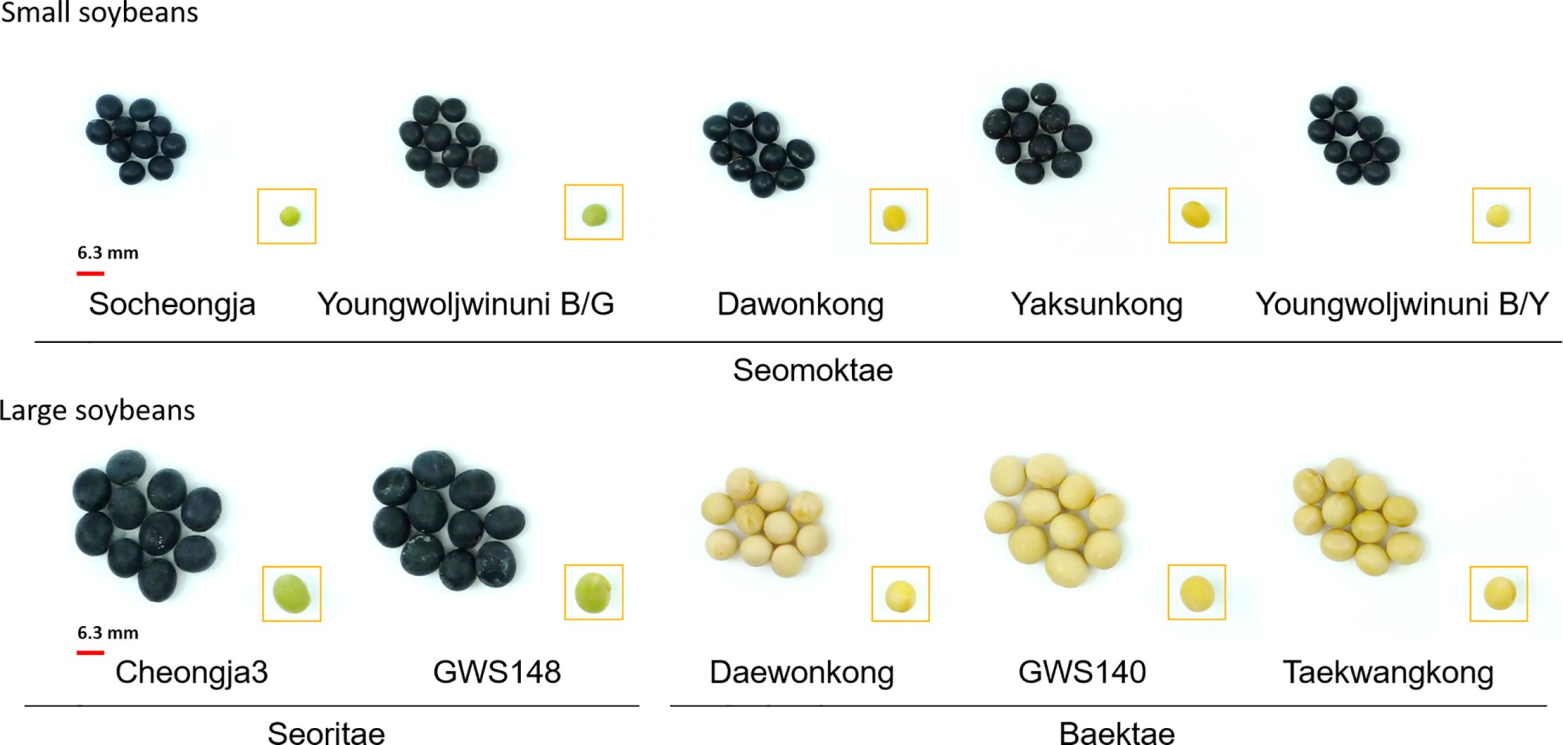
The visual characteristic of small-seeded and large-seeded soybeans. The boxes represent cotyledons of soybeans with the seed coat removed. Small-seeded soybeans: Socheongja (black seed coat / green cotyledon); Youngwoljwinuni B/G (black / green); Dawonkong (black / yellow); Yaksunkong (black / yellow); Youngwoljwinuni B/Y (black / yellow). Large-seeded soybeans: Cheongja3 (black / green); GWS148 (black / green); Daewonkong (yellow / yellow); GWS140 (yellow / yellow); Taekwangkong (yellow / yellow).

Soybeans are important nutrient-rich leguminous crops that are grown worldwide, particularly in Southeast Asia. In South Korea, where the supply of protein and fat is insufficient, soybean has been used as a primary source for several traditional fermented foods, which represent significant sources of nutrients for the indigenous population. On the basis of their use, Korean soybeans are classified as baektae [*Glycine max* (L.) Merr.], seoritae (*Glycine max* L.), and seomoktae (*Rhynchosia nolubilis*) [1]. Baektae cultivars have a white and yellow seed coat and are among the most highly cultivated soybeans in South Korea. The seed coat of seoritae cultivars is black, while its cotyledon is greenish in color and contains a range of functional substances, notably B vitamins, including niacin. The seed coat of seomoktae varieties is also black; however, in these cultivars, the cotyledon is yellow or green and the plants are characterized by a higher abundance of isoflavones compared with other cultivars of soybean. In general, baektae and seoritae have large (≥6.3 mm) seeds, whereas seomoktae have small seeds (≤6.3 mm) (Fig 1) [2].

The consumption of soybean-containing foods has been found to be effective in reducing the incidence of breast and prostate cancers and are effective to cardiovascular disease and menopausal symptoms [3]. These beneficial effects are attributable to the action of various bioactive compounds in soybeans, notably isoflavones [4–8].

Both soybean seeds and sprouts are used in a variety of foods; the nutritional value of sprouts is enhanced by germination [4, 7, 9]. The process of germination is characterized by the reactivation of seed metabolites, culminating in the emergence of the radicle and plumule [7], and is dependent on both internal and external environments, the most important of the latter of which are temperature, water, air, and light conditions. Among these, light is a significant factor for crop growth and development, with both light intensity and light quality regulating plant development and photosynthetic efficiency [10]. Compared with non-germinated soybean seeds, soybean sprouts contain a greater diversity of functional materials, and during the germination process, there are significant increases in the amounts of isoflavones, l-ascorbic acid, and phenolic compounds [6, 8].

Isoflavones, which are the primary physiologically active substance in soybeans, are members of phytoestrogen family that are structurally similar to the hormone estrogen [7], and can be divided into four groups (aglycones, glucosides, malonylglucosides, and acetylglucosides), each of which is classified into three different monomers [6, 11].

The most important isoflavones are the three glucosides genistin, daidzin, and glycitin [7]; aglycones consist of the biologically active forms genistein, daidzein, and glycitein; malonylglucosides include 6ʹ-*O*-malonylgenistin, 6ʹ-*O*-malonyldaidzin, and 6ʹ-*O*-malonylglycitin; and acetylglucosides consist of 6ʹ-*O*-acetylgenistin, 6ʹ-*O*-acetyldaidzin, and 6ʹ-*O*-acetylglycitin. It has been reported that the isoflavones of soybeans occur primarily as aglycones, as a consequence of the hydrolysis of glucosides during fermentation, whereas in non-fermented foods, the percentage of glucosides is notably high [12]. In addition, the isoflavone content of soybeans can be elevated in response to various stress factors; for instance, heat treatment at 60°C for 1 h has been shown to increase isoflavone content compared with the control [11].

The black seed-coated cultivars seoritae and seomoktae have been found to contain higher polyphenol compounds than either aronia or blueberry [13]. Anthocyanins as the main subclass of phenolic compounds, which are widely distributed in different plants parts, including fruits, flowers, stems, leaves, and roots, are water-soluble flavonoid pigments that impart red, purple, and blue colors [13]. It has been reported that polyphenols inhibit oxidation, suppress tumor cell proliferation, scavenge free radicals [14], and prevent the development of various cardiovascular diseases [3, 15].

In this study, we analyzed the germination-related changes in isoflavone content, bioactive compounds, antioxidant activities, and anti-inflammatory effects of five small-seeded black soybean cultivars and five large-seeded (black and yellow) soybean cultivars in South Korea. In addition, we compared the nutritional value of seeds and sprouts.

## Materials and methods

### Plant material

The visual characteristics of the 10 soybean types used in the present study, all of which were provided by the Gangwondo Agricultural Research and Extension Services (Chuncheon-si, Gangwon-do, Korea), are shown in Fig 1. Small-seeded and large-seeded soybeans are classified according to size, with small-seeded soybeans being classed as seomoktae and large-seeded soybeans as seoritae and baektae. The class seomoktae is represented by the five cultivars Socheongja and Youngwoljwinuni B/G (black seed coat, green cotyledon) and Dawonkong, Yaksunkong, and Youngwoljwinuni B/Y (black seed coat, yellow cotyledon). Seoritae comprises the two cultivars Cheongja3 and GWS 148, and Daewonkong, GWS 140, and Taekwangkong are the three baektae cultivars.

Soybeans were germinated at the Gangneung-Wonju National University in Gangneung, South Korea. The seeds were initially sterilized by immersing in 70% ethanol for 1 min, followed by immersion in a 2% sodium hypochlorite solution for 10 min with gentle shaking. Thereafter, the seeds were washed three times with sterilized water and then soaked in water for 5 h. The soaked seeds were then drained and cultured in Petri dishes containing 25 mL of Murashige and Skoog solid medium. The seed were subsequently germinated for 7 days at 25°C under two different conditions (illumination with incandescent light or maintained in darkness) in a tissue culture incubator (S1 Fig) (IB-05G; Jeio Tech, Korea). A total of 100 large-seeded soybeans were used for each cultivar, and 10 seeds and sprouts germinated under light and dark conditions on 0, 1, 3, 5, and 7 days were used for analysis. A total of 200 small-seeded soybeans germinated, and 20 germinated seeds and sprouts under each germination condition were used for the analysis.

### Chemicals

All chemicals, including solvents, used in the present study were of analytical grade. Isoflavones standards, including daidzein, glycitein, genistein, daidzin, glycitin, and genistin, DPPH (2,2-Diphenyl-1-picrylhydrazyl) free radical (90% purity), ABTS (2,2'-azino-bis-3-ethylbenzo-thiazoline-6-sulfonic acid), Folin-Ciocalteu’s phenol reagent, and sodium carbonate were purchased from Sigma-Aldrich (St. Louis, Mo., USA). The other solvents used in HPLC analysis were purchased from Daejung Chemicals (Siheung, Korea).

### Sample preparation

Sprouts of the 10 soybean cultivars were collected and frozen at -80°C in transparent polyethylene plastic bags sealed for later use. Samples were freeze-dried and ground into a fine powder using a mortar and pestle. Powdered samples (100 mg) were extracted with 1 mL of 70% ethanol for 12 h at 30°C in a water bath. After centrifugation at 26,712 × *g* for 15 min, the supernatant was filtered through a 0.22-μm syringe filter and used for analysis.

### Determination of isoflavones monomers

The determination of isoflavone monomers was performed as previously described by C-S Kim and Y-S Lee [16]. A 0.1 g sample of freeze-dried material was mixed with 1 mL of 70% methanol solution and extracted. The mixture was centrifuged and used for HPLC analysis.

The analysis of isoflavone monomers was conducted using a Shimadzu Prominence HPLC system (Shimadzu, Kyoto, Japan) equipped with a diode array UV-vis detector for monitoring at 280 nm. Compounds were separated using a C18 column (Shimadzu, Kyoto, Japan; 250 × 4.6 mm, 5 μm). Binary gradient elution was performed using solvent A (water containing 0.1% formic acid) and solvent B (acetonitrile containing 0.1% formic acid), which were delivered at a flow rate of 0.7 mL/min as follows: 0 min, 12% B; 20 min, 30% B; 50 min, 80% B; 53 min, 80% B; 54 min, 88% B; and 60 min, 88% B. The injection volume was 10 μL, and the column temperature was 40°C.

### Determination of total phenolic content

Total phenolic content was determined using the Folin-Ciocalteu reaction method, with gallic acid used as a standard [17]. Sprout extracts from the 10 soybean cultivars were diluted to 10,000 μg·mL^-1^ with 70% ethanol, and a 100-μL aliquot of each extract was mixed with 50 μL of Folin-Ciocalteu reagent for 3 min using a vortexer. Thereafter, 300 μL of 20% Na_2_CO_3_ solution was added to samples in a 1.5 mL micro tube, and the samples were reacted for 15 min at room temperature (23°C). The reaction solution (200 μL) was dispensed into 96-well plates. The absorbance of samples was measured at 738 nm using a microplate photometer (Multiskan FC; Thermo Scientific, MA, USA). Concentrations were determined using a calibration curve (R^2^ = 0.9964) generated using gallic acid and were expressed as mg gallic acid equivalents (GAE)·g^-1^ dry weight (DW).

### Determination of total flavonoid content

Total flavonoids were determined according to a method previously reported by R Re, N Pellegrini, A Proteggente, A Pannala, M Yang and C Rice-Evans [18] with modifications. Sprout extracts of the 10 soybean cultivars were diluted to 10,000 μg·mL^-1^ with 70% ethanol, 500 μL of which was mixed with 100 μL of 10% aluminum nitrate and 100 μL of 1 M potassium acetate in 96-well plates. After a 40-min reaction at room temperature, the absorbance was measured at 405 nm using a microplate photometer (Multiskan FC; Thermo Scientific, MA, USA). Concentrations were determined with reference to a calibration curve (R^2^ = 0.9998) generated using quercetin, and are expressed in terms of mg quercetin equivalents (QE)·g^-1^ DW.

### Antioxidant activities determined by the DPPH and ABTS methods

Extracts of the sprouts of the 10 soybean cultivars were diluted to 10,000, 5,000, and 2,500 μg·mL^-1^ with 70% ethanol. The DPPH radical scavenging assay was performed using a method previously described by R Re, N Pellegrini, A Proteggente, A Pannala, M Yang and C Rice-Evans [18] with modifications. A mixture of 100 μL of DPPH ethanol solution (0.15 mM) was added to an equal volume of sample extract, and after a 30-min reaction in the dark, the absorbance was measured at 517 nm, with ascorbic acid being used as a standard.

The ABTS radical scavenging activity assay was conducted according to a previously reported method YG Ku, DH Kang, CK Lee, SY Lee, CS Ryu, DE Kim, et al. [19] with modifications. The ABTS solution was dissolved in distilled water to a concentration of 7.4 mM and then mixed in a 1:1 ratio with 2.45 mM potassium persulfate to produce the ABTS radical cation (ABTS+). This solution was reacted overnight and then diluted with phosphate-buffered saline (PBS). Absorbance was measured at 738 nm using a microplate photometer (Multiskan FC; Thermo Scientific, MA, USA), with ascorbic acid being used as a standard.

### Inhibition of nitric oxide (NO) production

The murine macrophage RAW267.4 cell line was cultured in Dulbecco’s modified Eagle’s medium supplemented with 10% fetal bovine serum, 100 μg/L streptomycin, and 100 IU/mL penicillin at 37°C in a 5% CO_2_ atmosphere incubator. The RAW267.4 cells were seeded at a density of 20,000 cells/well in 96-well plates and incubated for 24 h at 37°C in a 5% CO_2_ atmosphere. Thereafter, 500 μL of extracts at different concentrations (11.1, 33.3, and 100 μg/mL) was added to each well of the microtiter plate, and after treatment for 2 h, cells were stimulated with 1 μg/mL of lipopolysaccharide (LPS) for 24 h.

Aliquots of supernatant (50 μL) obtained from the 96-well culture plates were transferred to a clean 96-well plate, followed by the addition 50 μL of Griess reagent (1% sulfanilamide + 0.1% naphthylenediamine dihydrochloride, 1:1) and incubation at room temperature for 10 min. The NO content in the supernatants was measured spectrophotometrically at 540 nm, with concentrations being determined using a standard nitrite concentration curve.

### Statistical analysis

The experiment was carried out using a completely randomized design. Measurements and analyses were performed in triplicate. Statistical analysis was performed using ANOVA in SAS (version 9.1). The significance of each measurement was determined using Duncan’s multiple range test at a significance level of *p* ≤ 0.05. To investigate the relationship between groups, we visualized data using R (version 3.3.1) and performed correlation analysis (PerformanceAnalytics, https://cran.rstudio.com/bin/windows/contrib/3.3/PerformanceAnalytics_1.5.2.zip; corrplot, https://cran.rstudio.com/bin/windows/contrib/3.3/corrplot_0.84.zip).

## Results and discussion

### Isoflavone concentrations during germination of soybeans of different sizes under light and dark conditions

**Fig 2:**
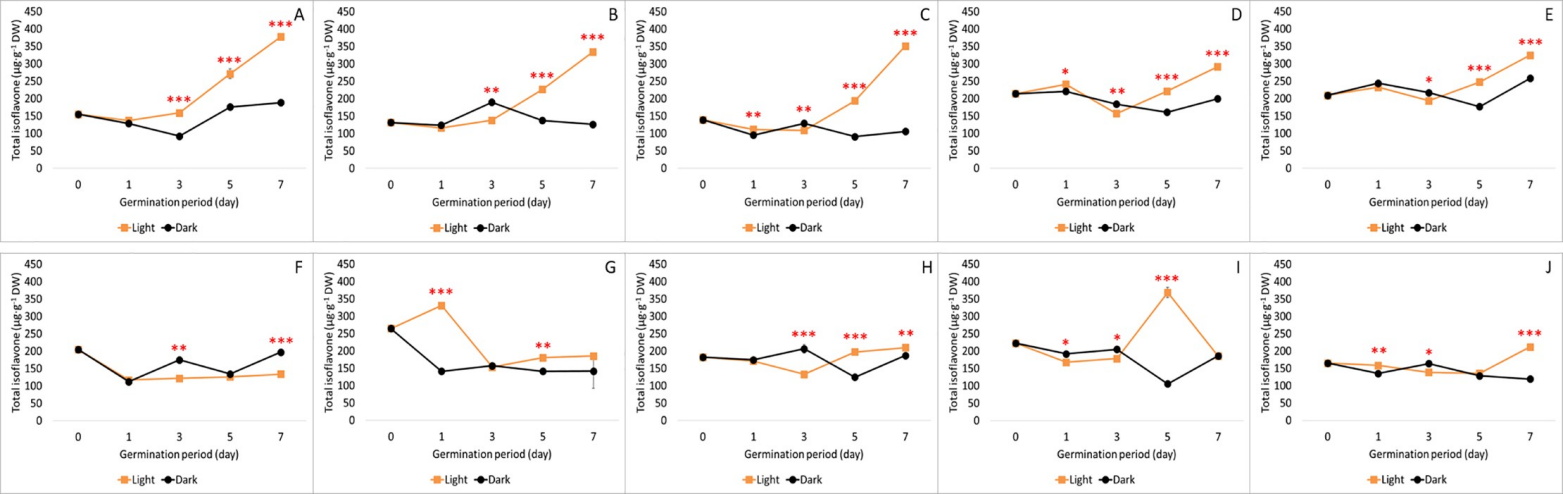
Changes in total isoflavones of small-seeded (A–E) and large-seeded (F–J) soybeans during germination under light and dark conditions. Values are the mean of three replicate determinations (n = 3) ± standard deviation. Statistical significance was shown by a t-test between the germination under light and dark conditions at the each time point (*** *p* < 0.001, ** *p* < 0.01, * *p* < 0.05). A, Socheongja; B, Youngwoljwinuni B/G; C, Dawonkong; D, Yaksunkong; E, Youngwoljwinuni B/Y; F, cheongja3; G, GWS 148; H, Daewonkong; I, GWS 140; J, Taekwangkong.

Among the four classes of isoflavones, we analyzed the content of the primary bioactive substances glucosides and aglycones. Fig 2 shows the total content of glucosides (daidzin, glycitin, and genistin) and aglycones (daidzein, glycitein, and genistein). We found that total isoflavone content showed differing trends among cultivars, depending on germination period. During early germination, large-seeded soybeans were observed to contain large amounts of isoflavones (172–273 μg/gDW), whereas small-seeded soybeans contained smaller amounts (126–205 μg/gDW). However, there was no differences in the color of seed coats or cotyledons. After 7 days of germination, we recorded the increase in the total isoflavone content of all cultivars, particularly among the small-seeded soybeans (Fig 2). Furthermore, total isoflavone content was found to be significantly higher in soybean sprouts (300–385 μg/gDW) germinated under light conditions than in those germinated under dark conditions (128–211 μg/gDW) (*p* < 0.001; Fig 2).

These observations are consistent with the findings of Lee et al., who found that isoflavone content increased with progression of the germination period, and were approximately 1.5-fold higher in the hypocotyl region than in the cotyledon [11]. Isoflavone content has also be shown to vary markedly depending on cultivar and have been reported to be influenced by genetic factors [11, 20].

In the present study, we found that the germination environment had differing effects on increases in the isoflavone content of large-seeded soybeans during the germination process. In Cheongja3, the isoflavone content increased when germinated under dark conditions, whereas in Daewonkong and Taekwangkong, it increased after 7 days of germination under light conditions. Overall, however, with respect to isoflavone content, light conditions were found to have less pronounced effects on large-seeded soybeans than on small-seeded soybeans.

Although black-coated soybeans have previously been reported to have lower isoflavone content than those with yellow seed coats [20], we detected no clear association between seed coat color and isoflavone content in the present study. We did, however, find that the content of isoflavones in both small-seeded and large-seeded soybeans were depended on the seed stage of the cultivar prior to germination and the increase in total isoflavone content was higher in small-seeded soybeans during germination (Fig 2). In this regard, a previous study has reported that isoflavone content differed according to soybean size, with the synthesis and accumulation of isoflavones being related to the size of embryos and cotyledons [21].

Among the isoflavones, notable changes in glucoside content was detected in all cultivar types (seomoktae, seoritae, baektae), with daidzin content being the highest, followed by glycitin and genistin (S2 and S3 Figs). Generally, we observed that in small the soybeans assessed, glucoside content tended to increase as the germination period progressed, whereas it remained constant or even decreased in large-seeded soybeans (Cheongja3, GWS 148, and Taekwangkong; S2 Fig). In particular, in the Dawonkong, Socheongja, and Youngwoljwinuni B/Y cultivars, we found that compared with early germination, glucoside content increased by more than 33-fold after 7 days of germination. Moreover, small-seeded soybeans germinated under light conditions showed highly significant differences (*p* > 0.001). Similarly, it has been reported that when several soybean cultivars were germinated, the isoflavone content increased by 20%–30% after 1–2 days of germination, and then gradually decreased [21]. It is also known that isoflavone content differs in different parts of germinated soybeans.

Previous studies [15] have shown that soybean sprouts contain more isoflavones than seeds, which can be attributed to the fact that seed germination increases the synthesis of isoflavones, and glycones are released from the isoflavone conjugates stored in the seed. In particular, isoflavones accumulate in the roots of germinating seeds, wherein they serve to inhibit phytopathogenic infections and are involved in the interaction between plants and microorganisms, thereby influencing the formation of soybean root nodules by nitrogen-fixing bacteria [22, 23].

With respect to aglycones, we observed an apparent increase in the content of these isoflavones in large-seeded soybeans, and after 7 days of germination, Cheongja3 contained higher amounts of aglycones (110 μg/g DW) than of glucosides (87 μg/g DW; S2 and S3 Figs). We detected high concentrations of genistein, which is associated with the pharmacological properties of soybeans [11, 20], in large-seeded soybeans and these increased with progression of the germination period, notably in Cheongja3. In South Korea, small-seeded black soybeans, which have low isoflavone contents, are used for medicinal purposes, suggesting that the pharmacological properties of soybeans are not associated with an increase in total isoflavone content [20]. In contrast, it has been reported that these properties are conferred by genistein and daidzein, which have been identified as pharmacological components of isoflavones [11, 15]. In this regard, aglycones are characterized by their bioavailability, as these forms of isoflavones can be readily absorbed in the human intestine, whereas other forms of isoflavones necessitate further hydrolysis [9, 12].

### Total phenolic and flavonoid content and antioxidant activities during germination of soybeans of different sizes under light and dark conditions

**Fig 3:**
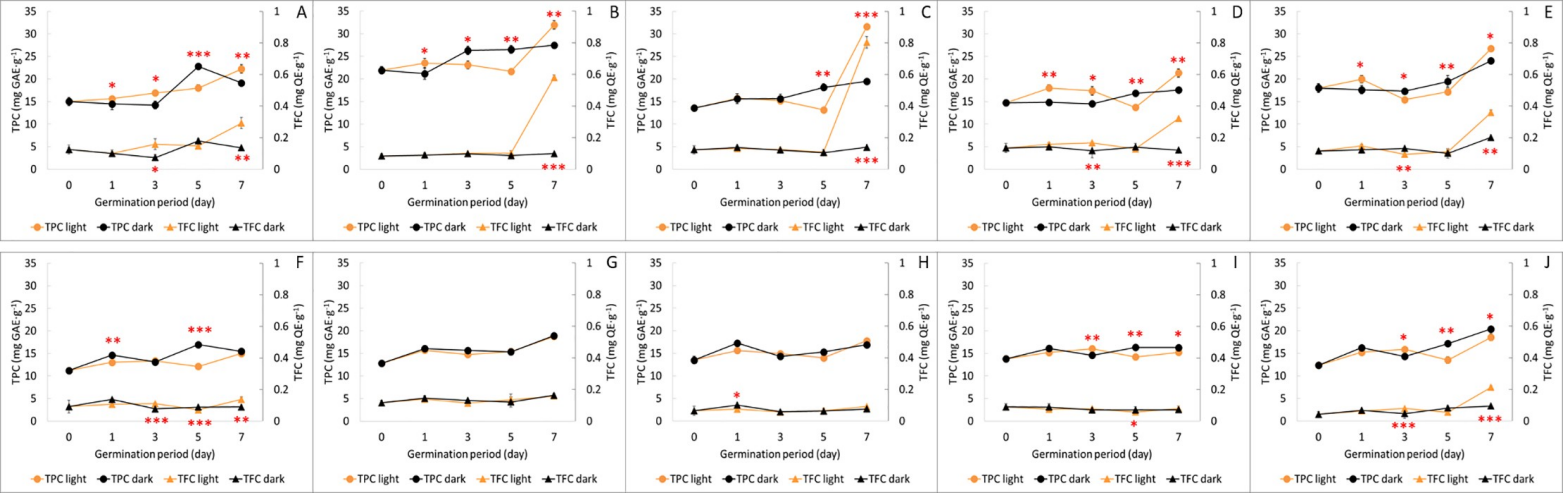
Changes in the total phenolic content and total flavonoid content of small-seeded (A–E) and large-seeded (F–J) soybeans during germination under light and dark conditions. Values are the mean of three replicate determinations (n = 3) ± standard deviation. Statistical significance was shown by a t-test between the germination under light and dark conditions at the each time point (*** *p* < 0.001, ** *p* < 0.01, * *p* < 0.05). A, Socheongja; B, Youngwoljwinuni B/G; C, Dawonkong; D, Yaksunkong; E, Youngwoljwinuni B/Y; F, Cheongja3; G, GWS 148; H, Daewonkong; I, GWS 140; J, Taekwangkong. GAE, gallic acid equivalents; QE, quercetin.

At the seed stage, the total content of phenolic compounds (TPC) was found to be slightly higher in small-seeded soybeans (13.6–22.5 mg GAE/gDW) than in large-seeded soybeans (10.9–13.2 mg GAE/gDW) and showed a similar tendency during the period of germination (Fig 3). After 7 days of germination, the TPC of small-seeded soybeans had increased to 22.7–32.8 mg GAE/gDW and 18.3–27.4 mg GAE/gDW under light and dark conditions, respectively. Germination under light conditions further increased the TPC of soybean sprouts. In large-seeded soybeans, the TPC of soybeans germinated for 7 days under light and dark conditions increased slightly to 14.9–18.7 mg GAE/gDW and 15.5–20.4 mg GAE/gDW, respectively. Unlike small-seeded soybeans, the TPC was higher in soybean sprouts germinated under dark conditions. As in the case of total isoflavones, the total phenolic content did not vary according to the seed coat and cotyledon color.

A previous comparison of soybean types undertaken by Jeong et al. (2017) indicated that the TPC was higher in the small-seeded seomoktae soybeans (275.8 ± 5.3 mg GAE/gDW) than in the large-seeded seoritae soybeans (255.1 ± 7.5 mg GAE/gDW), and consistently we found that at the seed stage, seomoktae cultivars had higher TPC content than seoritae cultivars, and that TPC increased with the progress of germination.

In small-seeded soybeans, the total flavonoid content (TFC) tended to be similar to the TPC and increased slightly as the germination period progressed. After 7 days of germination, the soybean sprouts produced under light conditions showed a significantly higher TFC than the soybeans germinated under dark conditions (*p* < 0.001; Fig 3A–3E). In large-seeded soybeans, with the exception of GWS 140, the TFC increased slightly during the 7 days of germination (Fig 3F–3J).

Phenolics are plant secondary metabolites that play roles in a diverse range bioactive processes during plant growth [13], among which flavonoids are an important group of compounds that are widely distributed in fruits and vegetables. Flavonoids have beneficial effects in protecting the body from various diseases, including cancer and cardiovascular diseases [14]. These physiological benefits of flavonoids are generally associated with their antioxidant capacities and the ability to eliminate free radicals, and accordingly have considerable therapeutic potential.

Small-seeded black soybeans, which in Korea are noted for their medicinal properties, are characterized by a high antioxidant activity conferred by the presence of proanthocyanidins in the seed coat [5, 14, 24]. Proanthocyanidins, a class of polyphenols, are present in a variety of plants and play vital roles as antioxidants in response to damage to and disorders of various macromolecules and organelles caused by lipid peroxidation and peroxynitrite [24]. Black soybeans are known to contain four types of anthocyanins, namely, delphinidin-3-glucoside, cyanidin-3-galactoside, cyanidin-3-glucoside, and cyanidin-3-arabinoside, among which cyanidin-3-glucoside is a significant compound in both seomoktae and seoritae [13].

**Fig 4:**
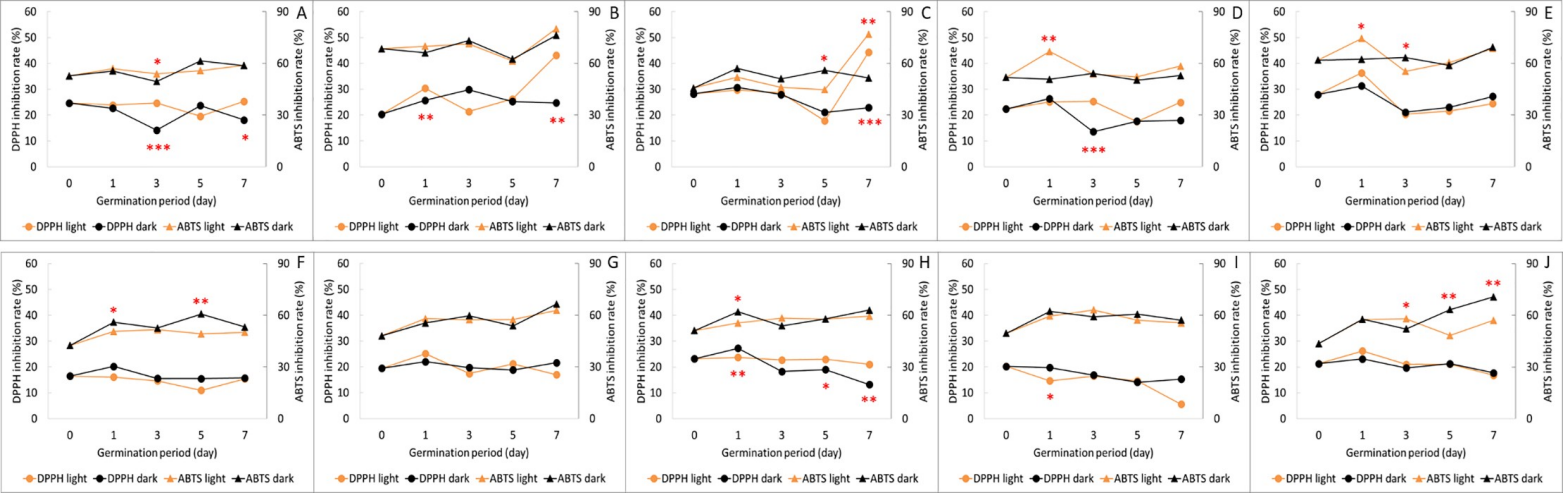
Changes in the total phenolic content, total flavonoid content, and antioxidant activities of small-seeded (A–E) and large-seeded (F–J) soybeans during germination under light and dark conditions. Values are the mean of three replicate determinations (n = 3) ± standard deviation. Statistical significance was shown by a t-test between the germination under light and dark conditions at the each time point (*** *p* < 0.001, ** *p* < 0.01, * *p* < 0.05). A, Socheongja; B, Youngwoljwinuni B/G; C, Dawonkong; D, Yaksunkong; E, Youngwoljwinuni B/Y; F, Cheongja3; G, GWS 148; H, Daewonkong; I, GWS 140; J, Taekwangkong. GAE, gallic acid equivalents; QE, quercetin.

In the present study, we confirmed antioxidant activity based on the scavenging abilities of DPPH and ABTS. In the case of the former, we detected an inhibition rate of 20.3%–28.2% in small-seeded soybeans and 16.5%–23.3% in large-seeded soybeans in the seed state (Fig 4).

Among the small-seeded soybean cultivars germinated under light conditions, the Youngwoljwinuni B/G cultivar, in which the DPPH inhibition rate increased to 22%, showed the highest activity after 7 days of germination, followed by Dawonkong, Yaksunkong, and Socheongja. In contrast, the Youngwoljwinuni B/Y cultivar was characterized by a reduced DPPH inhibition rate. Furthermore, in all cultivars with the exception of Youngwoljwinuni B/G, we observed a decrease in the DPPH inhibition rates of soybean germinated under dark conditions (Fig 4A–4E). For large-seeded soybeans, although the degree of germination did not affect the increase in DPPH inhibition rate, we found that rates tended to decrease in all cultivars under dark conditions (Fig 4F–4J).

In small-seeded and large-seededr soybeans, we recorded ATBS inhibition rates 45.7%–68.5% and 42.5%–51.1%, respectively, at the seed stage, which were notably higher than those of DPPH (Fig 4). In the case of small-seeded soybeans, the inhibition rate was higher in soybean sprouts germinated under light conditions, whereas in large-seeded soybeans, we noted a higher inhibition rate of soybean sprouts germinated under dark conditions. These differences were found to be similar to the patterns observed for TPC, namely small-seeded soybeans had a high TPC when germinated under light conditions, whereas large-seeded soybeans had a high TPC under grown under dark conditions. These observations accordingly indicate that phenolic compounds may play a significant role in antioxidant activities.

### Relationships among isoflavones, TPC, TFC, and antioxidant activities according to soybeans size

**Fig 5:**
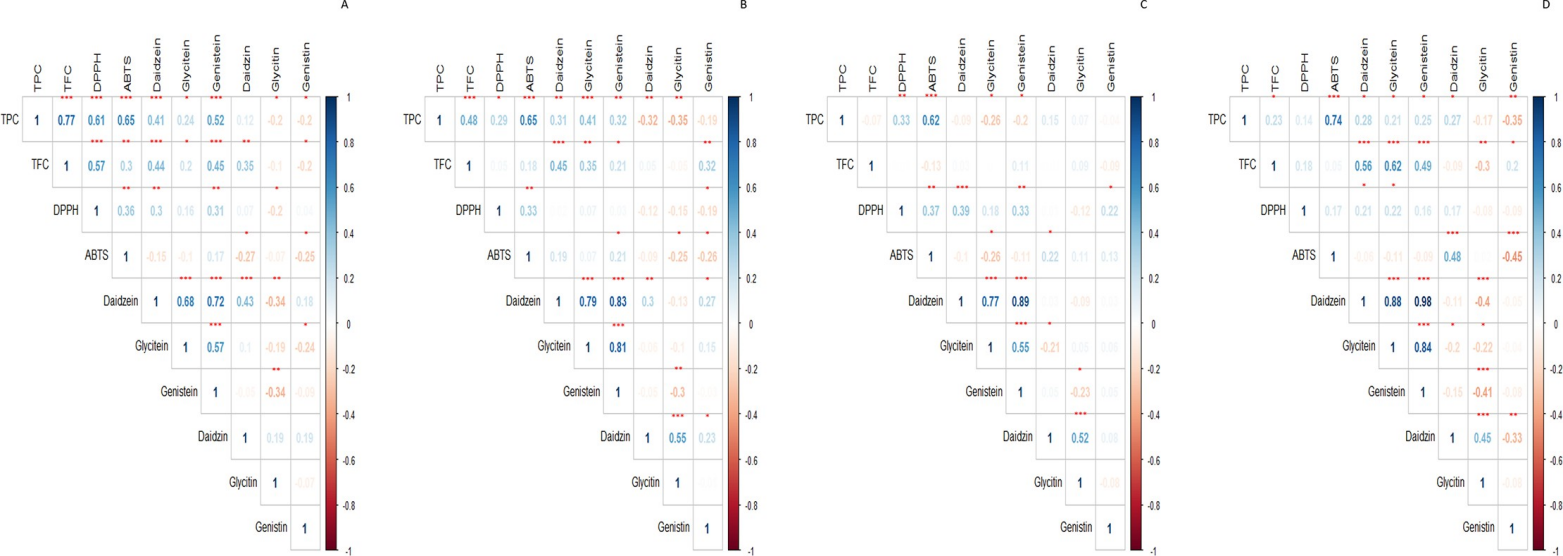
Correlations (R values) among isoflavones, TPC, TFC, and antioxidant capacity of small-seeded and large-seeded soybeans during germination under light and dark conditions. *p < 0.05; **p < 0.01; ***p < 0.001. A, small-seeded soybeans under light; B, large-seeded soybeans under light; C, small-seeded soybeans under dark; D, large-seeded soybeans under dark.

**Fig 6:**
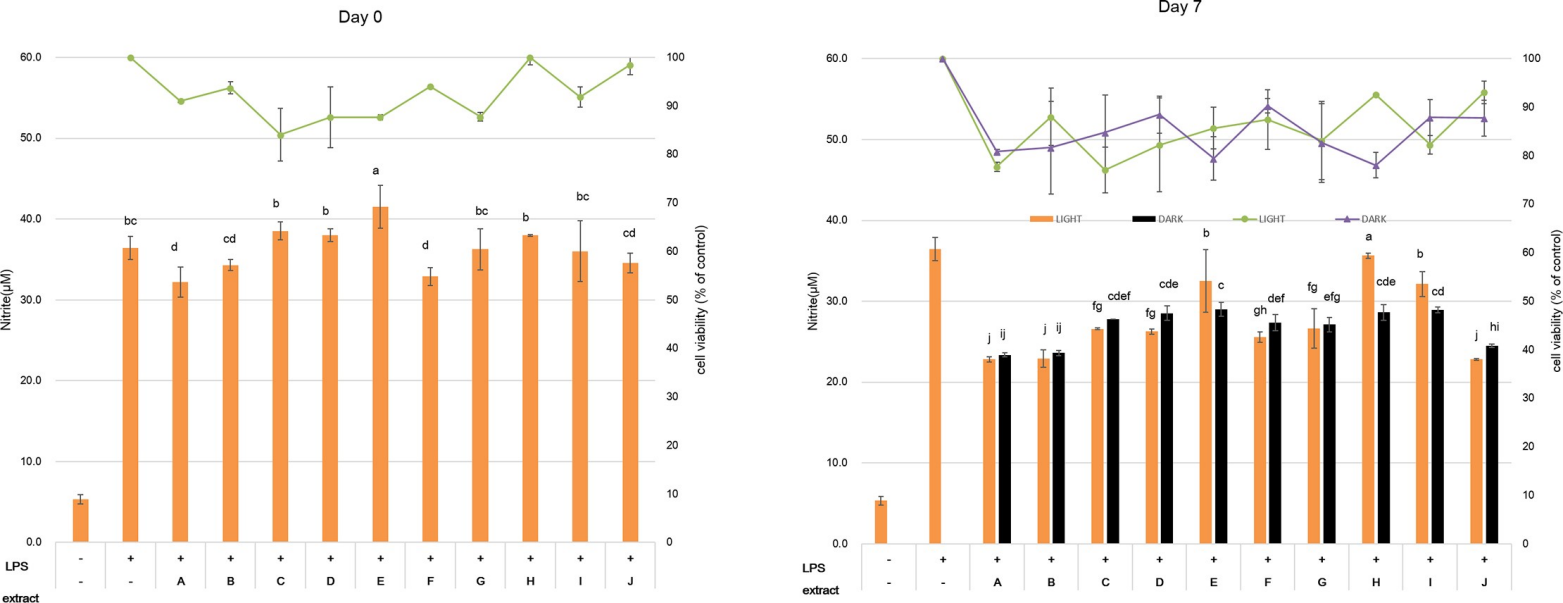
Effects of soybean extracts on nitric oxide production and cell viability of lipopolysaccharide (LPS)-activated RAW 264.7 cells. The cells were stimulated with LPS (1 μg/mL) only or with LPS plus extracts (100 μg/mL) of soybean cultivars. Values are the mean of three replicate determinations (n = 3) ± standard deviation. Bars denoted by different letters are significantly different (*p* < 0.05). Left, extract of 0-day soybeans after germination; Right, extract of 7-day soybeans after germination. A, Socheongja; B, Youngwoljwinuni B/G; C, Dawonkong; D, Yaksunkong; E, Youngwoljwinuni B/Y; F, Cheongja3; G, GWS 148; H, Daewonkong; I, GWS 140; J, Taekwangkong.

The correlations between assessed component and antioxidant activities for small-seeded and large-seeded soybeans are shown in Fig 6. For small-seeded soybeans germinated under light conditions, we found that the TPC showed a positive correlation (*p* < 0.001) with TFC, DPPH, ABTS, daidzein, and genistein, whereas the gluosides glycitin and genistin showed a negative correlation (*p* < 0.05; Fig 5A). The TFC showed a similar tendency to the TPC, although was not correlated with glucoside content (*p* < 0.05).

Compared with small-seeded soybeans, large-seeded soybeans tended to show less positive correlations (Fig 5B), among which the TPC showed positive (TFC, DPPH, ABTS, and aglycones) or negative (glucosides) correlations with other components. Soybean sprouts germinated under dark conditions showed lower correlations among components than those germinated under light conditions, although under both light conditions, aglycones (daidzein, glycitein, and genistein) were found to be highly correlated (*p* < 0.001). These results indicate that soybean sprouts germinated under light conditions are characterized by a high correlation among the components, and notably, in the case of small-seeded soybeans, light conditions affect the content of beneficial components.

### Anti-inflammatory effects of soybeans during germination

Under inflammatory conditions, lipopolysaccharide (LPS) activates RAW267.4 cells to generate nitric oxide (NO). To observe the anti-inflammatory effects of germinated soybeans, we accordingly used LPS to generate NO, which resulted in production of up to 36.4 μM NO in RAW267.4 cells. During the initial stage of germination (0 days), cells treated with all assessed cultivars at extract concentrations of 11.1, 33.3, and 100 μg/mL produced 33.1–41.8, 32.6–37.0, and 32.2–41.5 μM of NO, respectively (Fig 6 and S4 Fig). However, we observed no reduction in the NO content associated with the anti-inflammatory response to LPS.

With a progression of the germination period, all the soybean cultivars germinated under light and dark conditions showed anti-inflammatory effects by inhibiting the LPS-stimulated production of NO (S4 Fig). Treatment of cells with extracts obtained from 7-day germinated cultivars reduced the production of NO (Fig 6) depending on the soybean extract concentration, being reduced to below 25 μM in treatment with 33.3 μg/mL and 100 μg/mL soybean extracts. Among the small-seeded soybean cultivars, Socheongja was found to reduce NO production to below 25 μM at 33 μg/mL, whereas Socheongja and Youngwoljwinuni B/G had similar effects at 100 μg/mL. Among large-seeded soybeans, only Taekwangkong had a reductive effect when using a 100 μg/mL soybean extract. LPS, which is a component of the membrane structure of gram-negative bacteria and the endotoxin of pathogens, stimulates macrophages to induce various inflammatory mediators such as NO and prostaglandins [25, 26], the former of which is produced via the activity of inducible nitric oxide synthase. Although under normal conditions, NO plays a defensive role, and also acts as neurotransmitter and vascular regulator, at high concentrations, it can generate harmful substances such as peroxynitrite and nitrogen dioxide, which can result in DNA damage and the accumulation of harmful oxidants in cells [27]. In this regard, the genistein in soybeans has been reported to play an anti-inflammatory role [28, 29], the effect of which has been found to be concentration dependent. Similarly, it has previously been observed that among isoflavones, aglycones are more effective in inhibiting NO production than are other glucosides [30].

On the basis of an MTT (3-[4,5-dimethylthiazol-2-yl]-2,5-diphenyltetrazolium bromide) assay, we demonstrated that RAW 264.7 cells treated with 11.1 μg/mL extracts of all soybean cultivars showed over 80% cell viability (Fig 6), thereby confirming that soybean extracts are non-toxic to RAW 264.7 macrophages.

In this study, a comparison of the genistein contents of small-seeded and large-seeded soybeans (S3 Fig) indicated that the contents tended to be higher in large-seeded soybeans at 7 days from the beginning of germination. However, among the large-seeded soybeans, only Taekwangkong, in which the genistein content was lower than that in other varieties, showed an anti-inflammatory effect. In contrast, among the small-seeded soybeans, Socheongja and Youngwoljwinuni B/G showed good anti-inflammatory effects.

Contrary to the findings of a previous study by Kim and Kim (1997), we found no evidence of a positive correlation between the increase in isoflavones and pharmacological components in small-seeded soybeans. We did, however, find that the growth environment can have a marked effect on soybean constituents. For example, germination under light conditions was found to have a positive effect total isoflavones contents, whereas germination under dark conditions did not appear to have an appreciable effect on total isoflavones or anti-inflammatory properties. Moreover, we found that the contents of bioactive factors (TPC, TFC, and antioxidant activity) did not necessarily reflect the anti-inflammatory properties of the assessed soybean cultivars.

## Conclusion

In this study, we investigated changes in the various nutrient components of small-seeded (seomoktae) and large-seeded (seoritae and baektae) soybeans during the germination process. We found that total isoflavones were higher in the seeds of large-seeded soybeans, whereas that they had increased in small-seeded soybeans after 7 days of germination, particularly those germinated under light conditions. Although no differences were detected in the total isoflavone contents of small-seeded and large-seeded soybeans with respect to seed coat and cotyledon color, we found that most cultivars of small-seeded soybeans germinated under light conditions tended to have high TPC, TFC, and antioxidant activities. In contrast, in large-seeded soybeans, the germination environment generally did not significantly affect the TPC and TFC, and DPPH inhibition rates, although the ABTS activity of soybeans germinated under dark conditions was high. For all assessed cultivars germinated under both light and dark conditions, the anti-inflammatory effect of extracts obtained from germinated soybean sprouts were found to be superior to that of extracts obtained at the seed stage. Furthermore, we observed that the contents of bioactive factors (TPC, TFC, and antioxidant activity) did not necessarily reflect anti-inflammatory properties of the assessed cultivars. Collectively, these results indicate that the use of germinated soybeans would be nutritionally adequate when using soybeans in the food industry and that the enhancement of bioactivity under different germination environments could contribute to the selection of appropriate soybean cultivars. However, given that small-seeded and large-seeded soybeans differ with regard to their physiological activity depending on the germination environment, further studies are required to characterize the expression of related genes.

## Supporting information

S1 Fig Seed germination over 7 days of small-seeded and large-seeded soybeans. Small-seeded soybeans: B, Youngwoljwinuni B/G (black seed coat / green cotyledon); C, Dawonkong (black / yellow). Large-seeded soybeans: F, Cheongja3 (black / green); H, Daewonkong (yellow / yellow).
(DOCX)

S2 Fig Changes in isoflavone glucoside contents of small-seeded (A–E) and large-seeded (F–J) soybeans during germination under light and dark conditions. Values are the mean of three replicate determinations (n = 3) ± standard deviation. Statistical significance was shown by a t-test between the germination under light and dark conditions at the each time point (*** p < 0.001, ** p < 0.01, * p < 0.05). A, Socheongja; B, Youngwoljwinuni B/G; C, Dawonkong; D, Yaksunkong; E, Youngwoljwinuni B/Y; F, Cheongja3; G, GWS 148; H, Daewonkong; I, GWS 140; J, Taekwangkong.
(DOCX)

S3 Fig Changes in isoflavone aglycones content of small-seeded (A–E) and large-seeded (F–J) soybeans during germination under light and dark conditions. Values are the means of three replicate determinations (n = 3) ± standard deviation. Statistical significance was shown by a t-test between the germination under light and dark conditions at the each time point (*** p < 0.001, ** p < 0.01, * p < 0.05). A, Socheongja; B, Youngwoljwinuni B/G; C, Dawonkong; D, Yaksunkong; E, Youngwoljwinuni B/Y; F, Cheongja3; G, GWS 148; H, Daewonkong; I, GWS 140; J, Taekwangkong.
(DOCX)

S4 Fig Effects of extracts of soybeans germinated for 1, 3, 5, and 7 days on nitric oxide production in lipopolysaccharide (LPS)-activated RAW 264.6 cells. RAW 264.7 cells were stimulated with 11.1, 33.3, and 100 μg/mL of soybean extracts. The results are expressed as the mean ± SD of three independent experiments. A, Socheongja; B, Youngwoljwinuni B/G; C, Dawonkong; D, Yaksunkong; E, Youngwoljwinuni B/Y; F, Cheongja3; G, GWS 148; H, Daewonkong; I, GWS 140; J, Taekwangkong.
(DOCX)

S5 Fig HPLC chromatogram of isoflavones standard and isoflavones present in dawonkong soybeans germinated for 7 days. (DOCX)
