# Supplementary material for: Effect of germination environment on the biochemical compounds and anti-inflammatory properties of soybean cultivars
Source: PLoS One. 2020 Apr 27;15(4):e0232159. doi: 10.1371/journal.pone.0232159 (PMC7185686; doi:10.1371/journal.pone.0232159)
Supplement: S1 Fig — Small-seeded soybeans: B, Youngwoljwinuni B/G (black seed coat / green cotyledon); C, Dawonkong (black / yellow). Large-seeded soybeans: F, Cheongja3 (black / green); H, Daewonkong (yellow / yellow). (DOCX) [file pone.0232159.s001.docx]

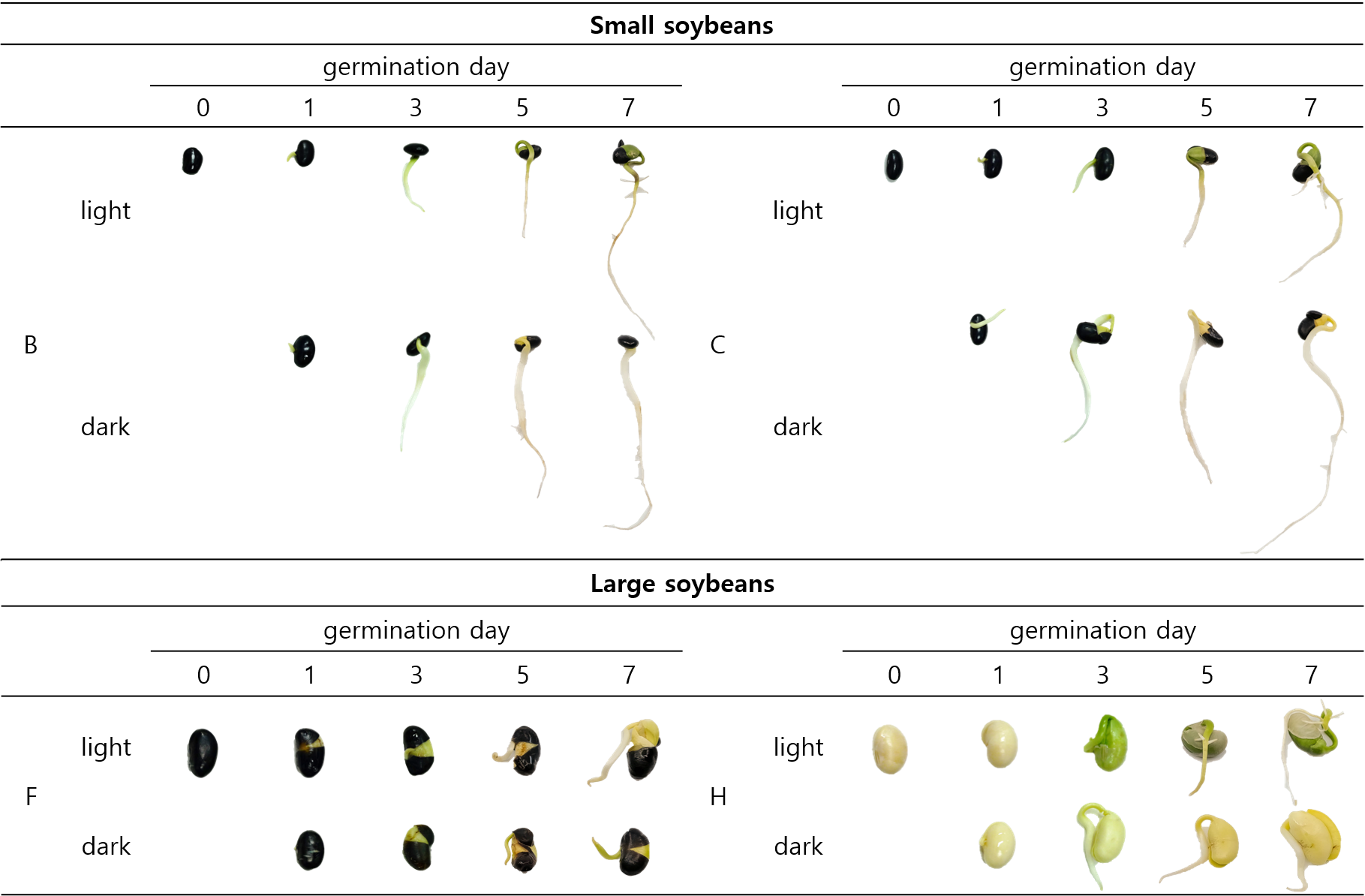


S1 Fig. Seed germination over 7 days of small and large soybeans. Small soybeans: B, Youngwoljwinuni B/G (black seed coat / green cotyledon); C, Dawonkong (black / yellow). Large soybeans: F, Cheongja3 (black / green); H, Daewonkong (yellow / yellow).
