# Supplementary material for: Effect of germination environment on the biochemical compounds and anti-inflammatory properties of soybean cultivars
Source: PLoS One. 2020 Apr 27;15(4):e0232159. doi: 10.1371/journal.pone.0232159 (PMC7185686; doi:10.1371/journal.pone.0232159)

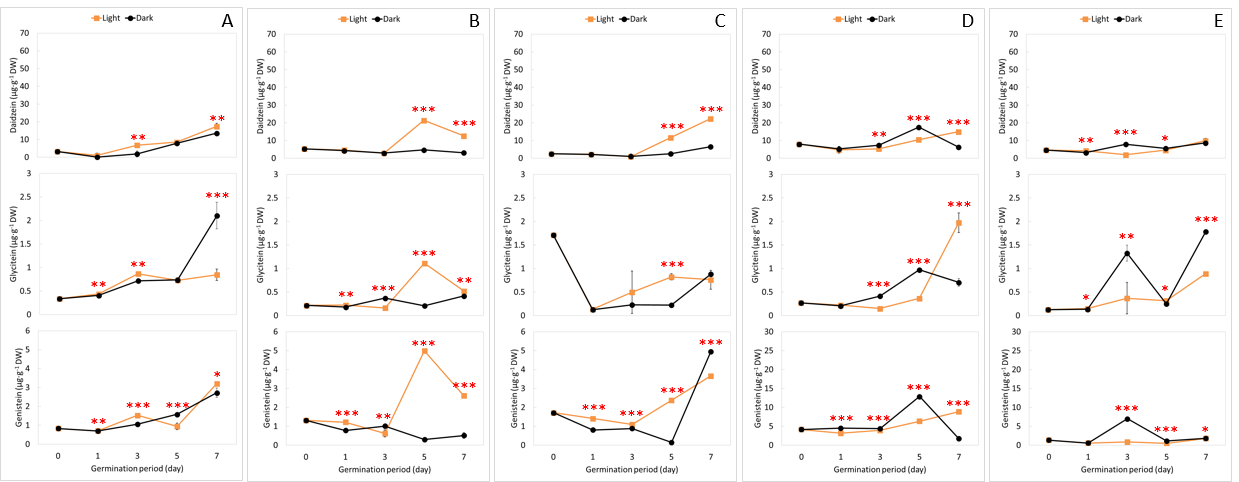


S3 Fig. Changes in isoflavone aglycones content of small (A–E) and large (F–J) soybeans during germination under light and dark conditions. Values are the means of three replicate determinations (n = 3) ± standard deviation. Statistical significance was shown by a t-test between the germination under light and dark conditions at the each time point (*** *p* < 0.001, ** *p* < 0.01, * *p* < 0.05). A, Socheongja; B, Youngwoljwinuni B/G; C, Dawonkong; D, Yaksunkong; E, Youngwoljwinuni B/Y; F, Cheongja3; G, GWS 148; H, Daewonkong; I, GWS 140; J, Taekwangkong.

S3 Fig continues


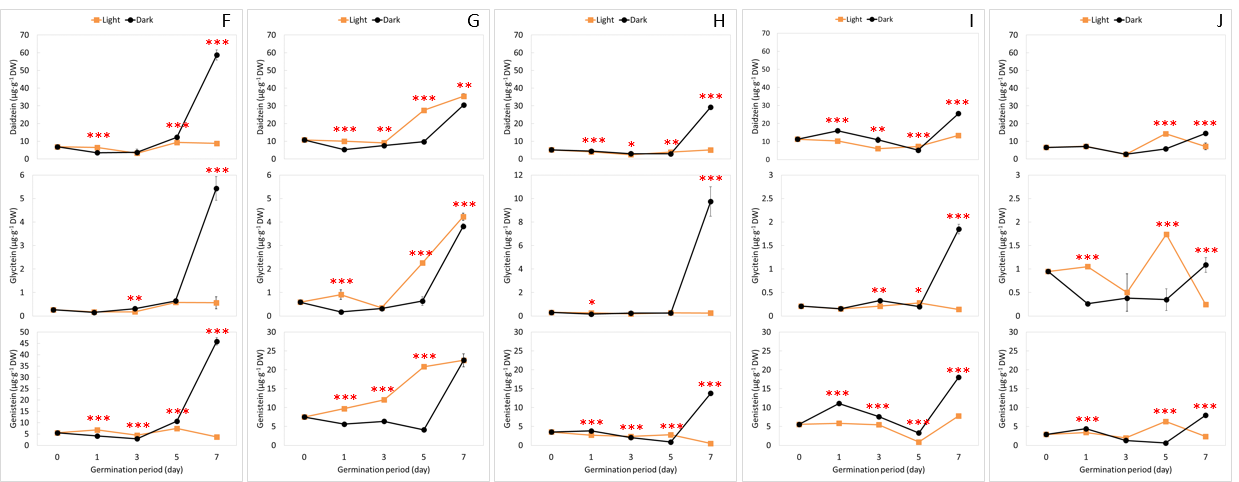

Supplement: S3 Fig — Changes in isoflavone aglycones content of small-seeded (A–E) and large-seeded (F–J) soybeans during germination under light and dark conditions. Values are the means of three replicate determinations (n = 3) ± standard deviation. Statistical significance was shown by a t-test between the germination under light and dark conditions at the each time point (*** p < 0.001, ** p < 0.01, * p < 0.05). A, Socheongja; B, Youngwoljwinuni B/G; C, Dawonkong; D, Yaksunkong; E, Youngwoljwinuni B/Y; F, Cheongja3; G, GWS 148; H, Daewonkong; I, GWS 140; J, Taekwangkong. (DOCX) [file pone.0232159.s003.docx]
