# Supplementary material for: Effect of germination environment on the biochemical compounds and anti-inflammatory properties of soybean cultivars
Source: PLoS One. 2020 Apr 27;15(4):e0232159. doi: 10.1371/journal.pone.0232159 (PMC7185686; doi:10.1371/journal.pone.0232159)
Supplement: S4 Fig — RAW 264.7 cells were stimulated with 11.1, 33.3, and 100 μg/mL of soybean extracts. The results are expressed as the mean ± SD of three independent experiments. A, Socheongja; B, Youngwoljwinuni B/G; C, Dawonkong; D, Yaksunkong; E, Youngwoljwinuni B/Y; F, Cheongja3; G, GWS 148; H, Daewonkong; I, GWS 140; J, Taekwangkong. (DOCX) [file pone.0232159.s004.docx]

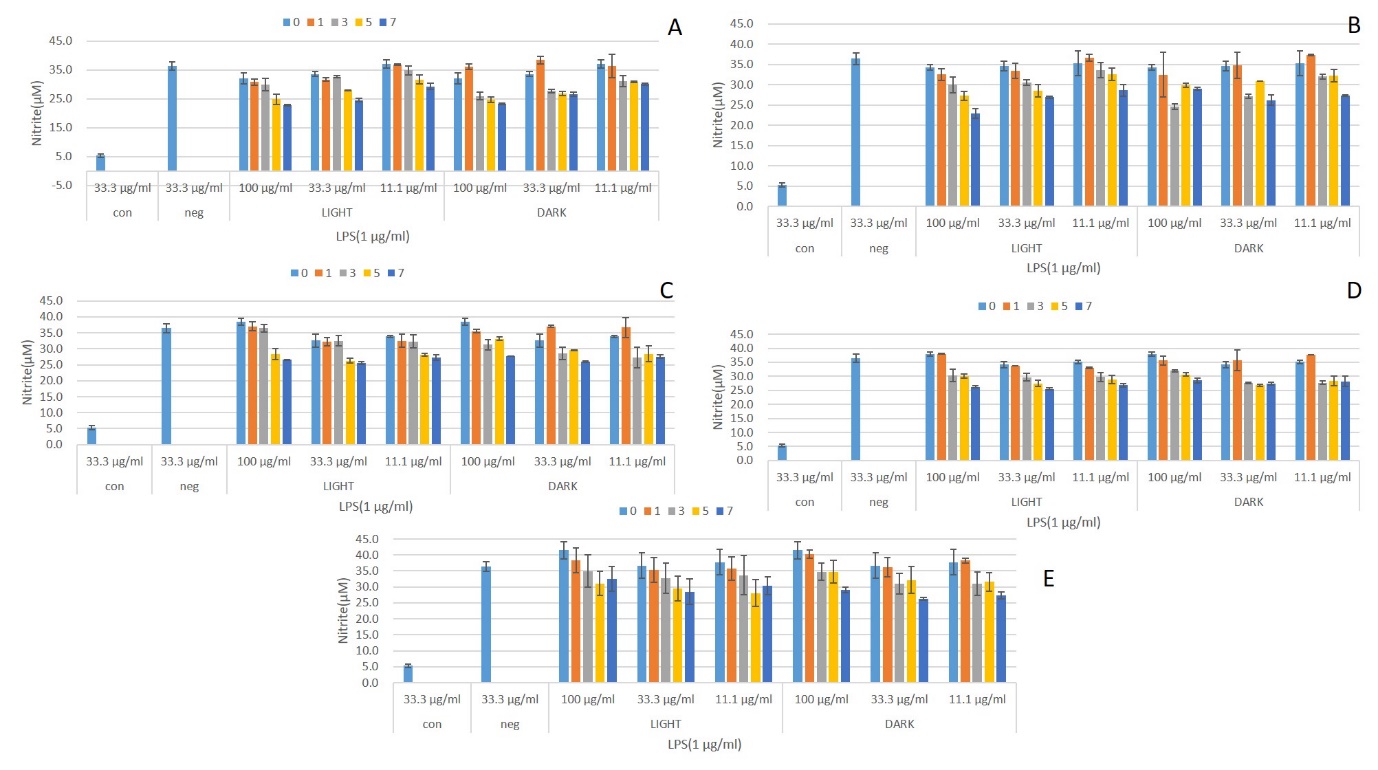


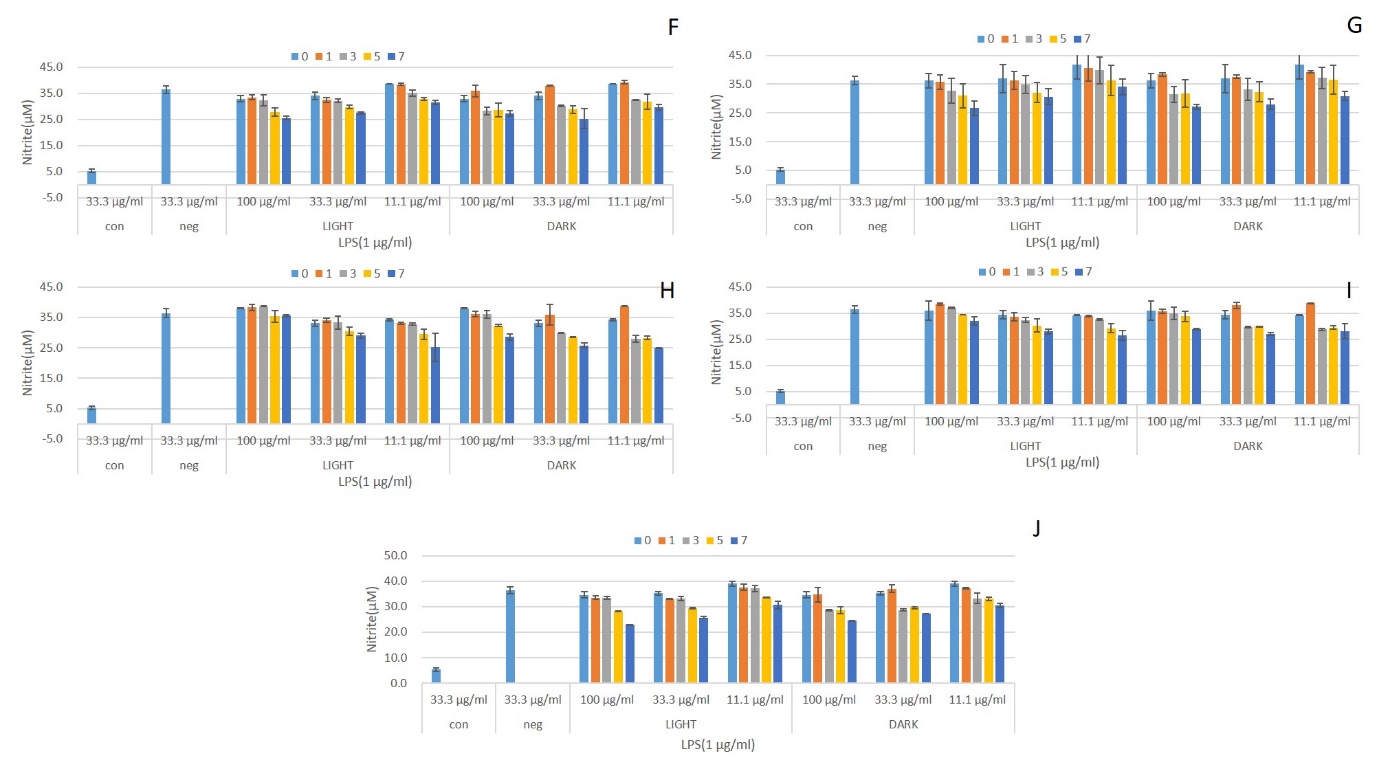


S4 Fig. Effects of soybeans extract germinated for 1, 3, 5, and 7 days on nitrite production of LPS-activated RAW 264.6 cells. RAW 264.7 cells were stimulated with 11.1, 33.3, and 100 μg/mL of soybeans extract. The results were expressed as mean±SD from three independent experiments. A, socheongja; B, youngwoljwinuni B/G; C, dawonkong; D, yaksunkong; E, youngwoljwinuni B/Y; F, cheongja3; G, GWS 148; H, daewonkong; I, GWS 140; J, taekwangkong.
