# Supplementary material for: Effect of germination environment on the biochemical compounds and anti-inflammatory properties of soybean cultivars
Source: PLoS One. 2020 Apr 27;15(4):e0232159. doi: 10.1371/journal.pone.0232159 (PMC7185686; doi:10.1371/journal.pone.0232159)
Supplement: S5 Fig — (DOCX) [file pone.0232159.s005.docx]

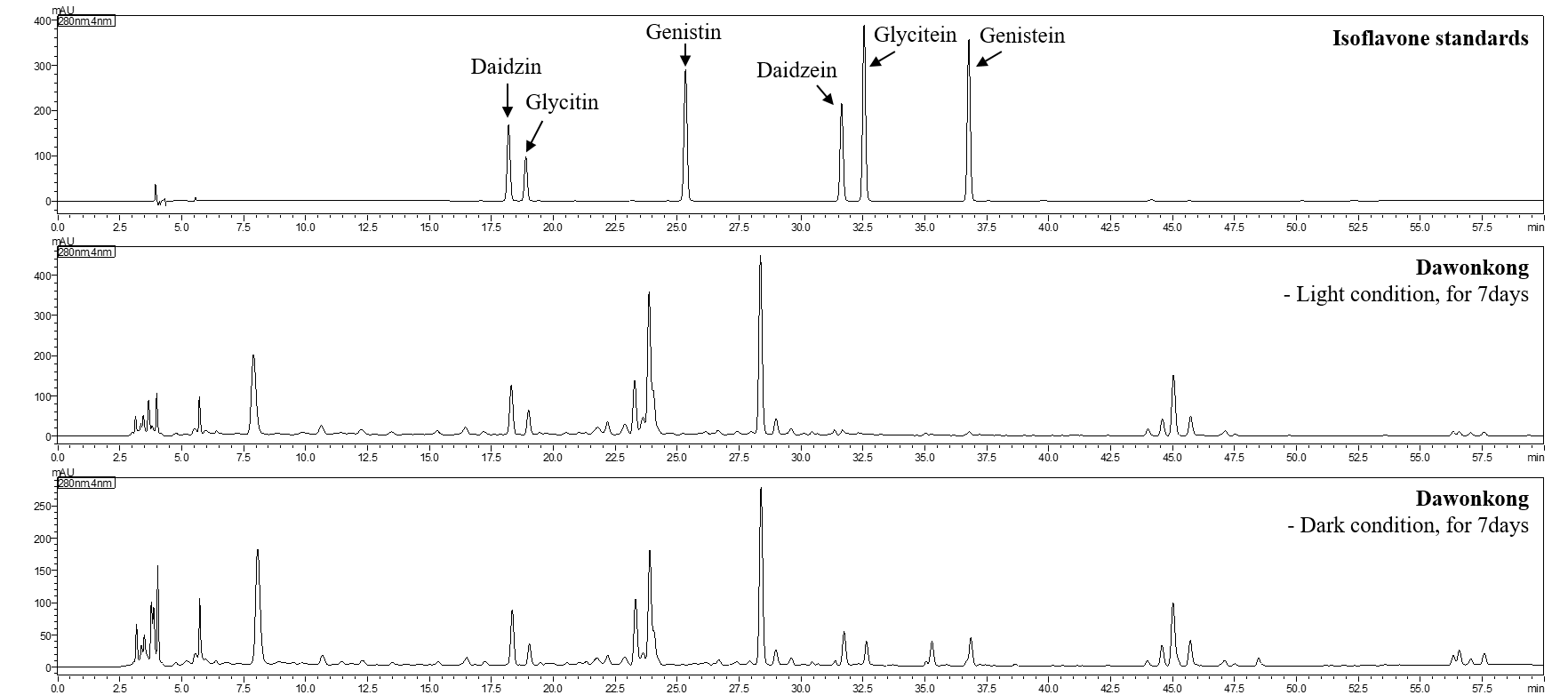


S5 Fig. HPLC chromatogram of isoflavones standard and isoflavones present in dawonkong soybeans germinated for 7 days.
